# From Registry to Reality: Opportunities to Enhance Post-market Surveillance of High-Risk Medical Devices: Comment on "Quality and Utility of European Cardiovascular and Orthopaedic Registries for the Regulatory Evaluation of Medical Device Safety and Performance Across the Implant Lifecycle: A Systematic Review"

**DOI:** 10.34172/ijhpm.9446

**Published:** 2025-11-29

**Authors:** Jessica N. Holtzman, Rita F. Redberg

**Affiliations:** Division of Cardiology, University of California, San Francisco, CA, USA.

**Keywords:** Medical Device Regulation, Post-market Surveillance, Cardiovascular Device, Safety, Real-World Evidence

## Abstract

Both the US Food and Drug Administration (FDA) and European Medical Device Regulation maintain the authority to require post-market surveillance for novel, high-risk medical devices. Yet, the current post-market surveillance system has several limitations. In their systematic review, Hoogervorst and colleagues highlight opportunities for improvement in the European medical device registries for the cardiovascular and orthopedic fields by standardised reporting of structural and methodological characteristics. Currently, there is heterogeneity in the governance, data collection, data reporting, and auditing of post-market surveillance registries, which limits their utility. Innovation in post-market surveillance mechanisms should include developing consensus around standardized safety outcomes by health condition, using artificial intelligence to identify patterns of harm in large or unstructured databases, continuing to invest in active surveillance networks to ensure the safety and effectiveness of high-risk medical devices, and educating physicians and care providers around adverse event reporting mechanisms. More resources and prioritization of post-market surveillance globally is needed to prioritize patient safety.

 In this issue of the *International Journal of Health Policy and Management*, Hoogervorst et al highlight opportunities for improvement in current European medical device registries.^1^ In a systematic review, the authors identified 46 active cardiovascular (assessing coronary artery stents and valvular prostheses) and orthopedic (assessing hip and knee prostheses) registries across Europe. The authors developed a list of best practice recommendations for registry maintenance and reporting based upon literature review, which was validated by a group of thirteen experts. This list contained 33 items across six quality domains including governance, data completeness, and quality assurance. The authors subsequently assessed publicly available material for each registry, including publications and annual reports, for each of the 33 prespecified quality metrics, noting that cardiovascular registries reported only a median of 33% of quality items, while 60% were reported by orthopedic registries. The authors highlighted that the lowest performing quality metrics across both sets of registries related to data quality and completeness, as well as safety and performance. Moreover, Hoogervorst and colleagues note that significant heterogeneity in quality, outcomes, and follow-up duration hinders the ability to pool outcomes, perform comparative effectiveness analyses, and provide timely detection of safety concerns. They suggest that standardization would improve this system.

 We agree that standardization of many aspects of medical device reporting would greatly strengthen regulatory systems in individual countries and expand the opportunities to share data internationally. Adverse event reporting would also be greatly strengthened by a higher engagement rate by healthcare providers and patients, easier reporting systems (such as mobile applications to report adverse events), and more transparency of data in reporting systems. For example in 2016, Dr. Hoonam Noorchasm worked with bipartisan legislators in the United States to introduce the “Medical Device Guardians Act” (H.R. 5404).^2^ Though this act would have added individual physicians and practitioners to the list of mandated “self-reporters”—in addition to the reporting requirements for hospitals and device manufacturers—it has not been adopted. Furthermore, the culture of safety in hospitals and postgraduate training could be strengthened to emphasize to trainees the key role of reporting safety events, and teaching the mechanics of reporting, which is currently not routinely covered.

 The growing volume of available medical devices within the international landscape of medical device regulation highlights critical challenges in establishing the safety and effectiveness of medical devices. Comparing post-market surveillance systems across regulatory environments provides opportunities for alignment and improvement. A recent estimate suggested that in 2019 in the US, more than $199.1 billion healthcare dollars were spent on medical devices and in vitro diagnostics.^3^ The Medical Device Amendments of 1976 implemented regulatory classifications based upon the device risk, categorizing class III devices as the highest risk. High-risk medical devices, though a minority (approximately 5%) of the total devices overseen by the US Food and Drug Administration (FDA), pose the greatest risk of bodily harm due to durable implantation or ability to sustain life.^4^ Novel devices, such as the recently approved coronary artery drug-coated balloon or transcatheter tricuspid valve repair and replacement systems, are classified as class III as they are determined to support or sustain human life or have the potential of significant harm. Though FDA authority to impose post-market restrictions and require periodic reporting of safety and effectiveness began as early as the Medical Device Amendments of 1976, there have been iterative changes over the course of nearly five decades to strengthen the comprehensiveness and rigor of post-marketing surveillance strategies. Likewise, the European Medical Device Regulation mandates that manufacturers of high-risk medical devices conduct post-market clinical follow-up that is proportionate to the device’s risk classification to evaluate its safety and efficacy.^5^ Despite these controls, international regulatory experts have noted the ongoing fallibility of the current post-market surveillance system for decades.^4,6,7^

 As in the European Union, there has been growing reliance upon post-market surveillance using device registries in the US. High-risk medical devices inherently pose a higher risk of bodily harm and are therefore subject to a higher evidentiary standard before entering the market, generally requiring more extensive supporting evidence prior to approval. However, clinical outcomes data are produced for a small minority of total medical devices, even-high risk devices.^8^ A growing number of pathways exist to facilitate the timely approval of medical devices in the US, including Premarket Approval, 510(k), De Novo, and Breakthrough Device pathways, each with its own specifications and requirements. Much has been written about the shortcomings of the 510(k) pathway, which necessitates only the demonstration of “substantial equivalence” to a previously marketed technology.^9^ The landmark Institute of Medicine report in 2010 called for the abandonment of the 510k process due to its inability to assure safety and effectiveness.^9^ Even via the more rigorous Premarket Approval pathway, generally used for the highest risk and most novel medical devices, data generation requirements are flexible and of inconsistent quality, often using surrogate end-points, with a minority of manufacturers required to generate randomized, blinded, controlled trials to demonstrate safety and efficacy.^8^ Though the nature of certain medical devices makes blinding or even randomization realistically challenging, in the absence of high-quality premarket data, the onus falls upon rigorous post-market data collection to monitor safety outcomes.^10^ Yet, the structure, content, and quality of these data registries remains highly variable.

 Hoogervorst and colleagues highlight heterogeneity in the governance, data collection, data reporting, and auditing or surveillance of post-market surveillance across many active European registries.^1^ In the US, the FDA relies upon a variety of passive mechanisms for post-market surveillance. Traditionally, the FDA has required that device sponsors report adverse safety events using a database entitled the Manufacturer and User Facility Device Experience (MAUDE).^11^ Though an important contributor to post-market surveillance, significant limitations have been identified with passive surveillance strategies, including delayed or absent reporting leading to missed safety events, inaccurate categorization of events, duplication of events, and lag time to detection of patterns of harm.^12-14^ Importantly, passive surveillance strategies rely upon physicians and patients to voluntarily report safety events via the manufacturer. Reports to MAUDE must then be reviewed and responded to in a timely fashion in order to identify safety concerns; from 2003-2007, far less than 50% of reports were documented to have been reviewed within 60 days of receipt.^15^

 Given these limitations, the FDA has supported the development of active surveillance strategies for medical devices. In 2007, the FDA announced the development of the Sentinel Initiative, a data-monitoring tool using electronic health data to generate real-world evidence for safety monitoring.^16^ Subsequently, in 2016, the Center for Devices and Radiologic Health developed the National Evaluation System for health Technology (NEST) for the purpose of collecting data from a consortium of hospitals and payors to actively assess safety outcomes of medical devices. After construction of the necessary cloud-based infrastructure for data management, NEST began active surveillance of two medical devices (a duodenoscope and a robot-assisted device involved in gallbladder removal) in December 2024, with no outcomes or safety reports yet publicly available. There are plans to expand the scope of analyses within the next five years, adding four devices and 10 million new patients per year.^17^

 Hoogervorst and colleagues note that the absence of a unique device identifier (UDI) code, or the failing to record UDI codes, limits both the comprehensiveness, as well as the comparability and compatibility of separate European device registries. This remains a key limitation in the US, as well.^7^ Though pharmaceutical claims list a standardized national drug code for each prescription medication that is dispensed, medical device claims and electronic health records still do not report UDIs for implantable medical devices in a consistent fashion. Consistent reporting of UDIs in claims data, as well as electronic health data, would significantly enhance capacity for active surveillance leading to early detection of adverse events, as well as flagging inconsistencies in utilization patterns or opportunities for more equitable implementation.

 Ongoing innovation and development of novel technologies gives us the opportunity to treat previously life-limiting illnesses with increasing success. But, with evolving technologies, it is our responsibility as members of the healthcare community to ensure that they are being deployed safely and responsibly. As the burden of evidence generation to assure safety and effectiveness shifts toward post-market surveillance, it is important that patients understand the current state of evidence when consenting to a therapy and are aware that data generation is ongoing. Physicians and healthcare providers should receive education around how to discuss the nascent state of evidence with patients in order to obtain informed consent, as well as how to report adverse events through mechanisms like MAUDE. As active surveillance systems are being developed, physician reporting of adverse events may serve as a temporary solution to draw attention to safety concerns, though is unlikely to fully address the limitations of current device registries. Development of mobile application for adverse event reporting could facilitate rapid, real-time reports. As Hoogervorst et al note, developing consensus around standardized safety outcomes by condition would allow for comparative effectiveness evaluation and increased consistency.^1^ Though still in its early stages, researchers may explore the role for using artificial intelligence to identify patterns in large datasets or to use natural language processing to extract device-related complications from unstructured clinical data. Of course, artificial intelligence models will only be as good as the data upon which they are trained, so prioritizing unbiased and complete data collection would be key. Additionally, medical education and medical culture must change to make the reporting of adverse events the standard of care by the profession. Lastly, ongoing legislative and financial commitment to supporting active surveillance of medical devices will be key to facilitate the expansion of initiatives like Sentinel and NEST.^14^ Given finite administrative resources, collective decisions need to be made in terms of the appropriate duration of post-market surveillance for a given medical device and when sufficient evidence has been collected to warrant the retirement of a given registry.

 Hoogervorst and colleagues underscore how heterogeneity in the structure and implementation of existing medical device registries in the European Union limits their utility, yet the lessons learned are applicable more universally. International regulators, manufacturers, and the academic community must continue to invest in coordinated and transparent post-market data collection. In doing so, this will ensure that novel technologies reach patients quickly, but do so in a safe, effective, and durable fashion.

## Disclosure of artificial intelligence (AI) use

 Not applicable.

## Ethical issues

 Not applicable.

## Conflicts of interest

 Rita F. Redberg receives grant support from the Arnold Ventures Foundation. Jessica N. Holtzman reports no financial or other disclosures.
